# SARS-CoV-2 in the Air Surrounding Patients during Nebulizer Therapy

**DOI:** 10.1155/2022/9297974

**Published:** 2022-09-17

**Authors:** Jostein Gohli, Arne Broch Brantsæter, Kari Oline Bøifot, Carola Grub, Beathe Kiland Granerud, Jan Cato Holter, Anne Margarita Dyrhol Riise, Madelen Foss Smedholen, Marius Dybwad

**Affiliations:** ^1^Norwegian Defence Research Establishment, P. O. Box 25, No. 2027 Kjeller, Oslo, Norway; ^2^Department of Infectious Diseases, Oslo University Hospital, P. O. Box 4956, Nydalen, No. 0424, Oslo, Norway; ^3^Norwegian National Unit for CBRNE Medicine, Oslo University Hospital, P. O. Box 4956, Nydalen, No. 0424, Oslo, Norway; ^4^Department of Analytics, Environmental & Forensic Sciences, King's College London, 150 Stamford Street, London SE1 9NH, UK; ^5^Institute of Microbiology, Norwegian Armed Forces Joint Medical Services, P. O. Box 25, No. 2027, Kjeller, Oslo, Norway; ^6^Department of Microbiology, Oslo University Hospital, P. O. Box 4950, Blindern, No. 0424, Oslo, Norway; ^7^Department of Nursing, Health and Laboratory Science, University College of Østfold, P. O. Box 700, No. 1757, Halden, Oslo, Norway; ^8^Institute of Clinical Medicine, University of Oslo, P. O. Box 1171, Blindern, No. 0318, Oslo, Norway; ^9^Oslo University Hospital, P. O. Box 4950, Nydalen, No. 0424, Oslo, Norway

## Abstract

Nebulizer therapy is commonly used for patients with obstructive pulmonary disease or acute pulmonary infections with signs of obstruction. It is considered a “potential aerosol-generating procedure,” and the risk of disease transmission to health care workers is uncertain. The aim of this pilot study was to assess whether nebulizer therapy in hospitalized COVID-19 patients is associated with increased dispersion of SARS-CoV-2. Air samples collected prior to and during nebulizer therapy were analyzed by RT-PCR and cell culture. Total aerosol particle concentrations were also quantified. Of 13 patients, seven had quantifiable virus in oropharynx samples, and only two had RT-PCR positive air samples. For both these patients, air samples collected during nebulizer therapy had higher SARS-CoV-2 RNA concentrations compared to control air samples. Also, for particle sizes 0.3–5 *µ*m, particle concentrations were significantly higher during nebulizer therapy than in controls. We were unable to cultivate virus from any of the RT-PCR positive air samples, and it is therefore unknown if the detected virus were replication-competent; however, the significant increase in smaller particles, which can remain airborne for extended periods of time, and increased viral RNA concentrations during treatment may indicate that nebulizer therapy is associated with increased risk of SARS-CoV-2 transmission.

## 1. Introduction

Since the World Health Organization (WHO) declared coronavirus disease 2019 (COVID-19), caused by the severe acute respiratory syndrome coronavirus 2 (SARS-CoV-2), a global pandemic on March 11^th^, 2020 [1], the virus has caused more than 418 million diseased cases and over 5.8 million deaths [2] worldwide.

The 2003 outbreak of SARS-CoV-1 and the ongoing MERS-CoV epidemic have demonstrated that health care workers (HCW) are at risk of nosocomial infection [3]. The level of environmental SARS-CoV-2 contamination, both in air and on surfaces, is significantly higher in hospitals as compared to other environments [4]. Unsurprisingly, COVID-19 has disproportionality affected HCW [5–8], resulting in reduced treatment capacity and additional strain on already overburdened health care systems [9]. Therefore, appropriate infection prevention and control measures such as personal protective equipment and vaccination of HCW are of utmost importance. However, while the majority of HCW in high-income countries are vaccinated, only 27% in Africa are fully vaccinated [10]. Vaccinations significantly reduce, but do not remove the risk of symptomatic infection [11, 12], asymptomatic infection [12, 13], transmission [12], or even severe illness [12, 13]. Therefore, to prevent HCW from infection with SARS-CoV-2, identifying high-risk procedures is important.

Aerosol-generating procedures (AGP) produce small respiratory particles. While no universally accepted list of AGP exists [14], the WHO includes procedures such as intubation, cardiopulmonary resuscitation, bronchoscopy, and surgery involving the use of high-speed devices [15]. The potential link between AGP and transmission of acute respiratory infection has long been debated [16–18], and the current pandemic has sparked further controversy [19, 20], including the potential risk of SARS-CoV-2 transmission to HCW [21–24]. Aerosol particles are very small particles that may stay suspended in the air for some time and have the ability to travel distances >2 meters [25]. The upper size limit of what constitutes an aerosol particle is context-dependent and in no way absolute—different operational definitions include particles of widely different sizes [26]. Since small aerosol particles can penetrate and circumvent surgical masks, AGP may necessitate the use of N95 or higher protection respirators and eye protection [19]. Optimally, AGP performed in patients with acute respiratory tract infections should be carried out in airborne infection isolation rooms with frequent air changes and negative pressure [27]. It is worth noting that the risk associated with AGP is not a simple function of the number of aerosol particles produced but is dependent on a number of factors that may contribute to transmission risks such as the size distribution of aerosol particles [28], the amount of force applied on air over a moist respiratory mucous membrane, and the virus load in the involved tissues [19].

Nebulizer therapy (NT) is often used in the treatment of patients with chronic obstructive pulmonary disease and to relieve symptoms in patients with acute pulmonary infection with cough and signs of obstruction. NT changes medication from liquid to an inhalable mist. NT is considered a “possible aerosol-generating procedure,” but whether NT represents an increased infection risk for HCW is still controversial [18, 19, 29]. Since aerosols are produced from medication during NT, and not from patient tissues, some argue that there is no associated increased risk of transmission [30]. However, the jet applied during NT can potentially also induce aerosol generation from infected tissue fluids [31], leading to room contamination by exhaled air, which was the likely driving factor in one hospital outbreak of SARS-CoV-1 in 2003 [32]. Therefore, it has been argued that NT is a significant risk factor for SARS-CoV-2 transmission [33, 34]. However, very limited evidence exists to support either view [35].

In this pilot study, we explored the impact of NT on the concentration level of total aerosol particles and SARS-CoV-2 in the air surrounding hospitalized COVID-19 patients receiving such treatment.

## 2. Methods

### 2.1. Study Participants

This pilot study was conducted at Oslo University Hospital (OUH) in collaboration with a prospective observational cohort study of hospitalized adults (aged ≥18 years) with COVID-19: “The Norwegian SARS-CoV-2 study – virological, clinical, and immunological characterization of inpatients during the COVID-19 outbreak” (ClinicalTrials.gov Identifier: NCT04381819 [36]). Thirteen COVID-19 patients admitted to the ward at the Department of Infectious Diseases, OUH were included. Five were female and eight were male, with a mean age of 55.3 years (min = 37, max = 81; Table 1). None of the participants were vaccinated for COVID-19. Informed consent was given by all patients or from next of kin if patients were incapacitated and therefore unable to give consent. The pilot study was approved by the Regional Committees for Medical and Health Research Ethics in South-Eastern Norway (reference no. 106624).

### 2.2. Collection of Air Samples

All patients were admitted to an airborne-precaution room with >22 exchanges of air/hour and negative pressure of 30 Pa with reference to the ordinary clinical areas of the ward. NT was given with Philips Respironics SideStream disposable kits (Respironics Respiratory Drug Delivery Ltd., Chichester, UK) with an oxygen flow of 8 L/min. The NT procedure lasted for approximately 10 minutes and consisted of inhalation of a mixture of 2.5 ml of salbutamol 1 mg/ml and 1 ml of ipratropium bromide 0.25 mg/ml.

Air samples from the isolation rooms were collected immediately prior to and during NT. Air sampling was conducted on average 13.4 (median = 12; range 7–23; Table 1) days after symptom onset. First, the air was sampled for 15 minutes prior to the administration of NT (hereafter referred to as the “control sample”). Then, a second 15-minute air sample was collected, starting from the beginning of the NT. Talking, mild coughing, severe coughing, laughing, and sneezing was recorded during air sampling (Table 1). Air samples were collected on electret filters with SASS3100 air samplers (300 L of air per minute; Research International, Monroe, WA, USA). The air samplers were placed at the foot end of the bed, at a height of 1.2 meters with a 45° downward angle to avoid deposition of larger respiratory droplets on the filters. The SASS inlet was cleaned with ethanol wipes before the filters were mounted. For both the control and the NT samples, two SASS air samplers were used in parallel–one filter was analyzed with RT-PCR and one with cell culture. Air filters for RT-PCR were transported on blue ice to the laboratory in 50 ml sterile vials containing 10 ml of NucliSENS lysis buffer (BioMérieux, Marcy-l'Étoile, France) and stored at −80°C until further processing. Air filters for cell culture were placed directly in 50 ml sterile vials containing 10 ml of Dulbecco's modified essential medium (DMEM, Sigma) with 1% penicillin/streptomycin/amphotericin B (PSA, Gibco) and transported to the lab on blue ice.

### 2.3. Quantification of Particle Concentrations

Particle concentrations were quantified using an AeroTrak 8220 (TSI, Shoreview, MN, US) optical particle counter (Model: 1300102), which was also placed at the foot end of the bed, at the same height as the air samplers. The AeroTrak was operated at a flow rate of 2.8 liters of air per minute (±5% accuracy) and binned particles as follows: 0.3–0.5, 0.5–1, 1–3, 3–5, 5–10, and >10 *µ*m. Due to equipment malfunction, we only retrieved particle count data for 12 of the 13 patients.

### 2.4. Processing and RT-PCR of Air Samples

Air samples were thawed and vortexed, before removing filters from the lysis buffer using sterile forceps and placing them in sterile syringes to extract the remaining liquid back into the lysis buffer vial. Before RNA isolation with NucliSENS magnetic extraction reagents (BioMérieux), an internal control (LightMix Modular EAV RNA Extraction Control, TIB-MOLBIOL, Germany) was added (1 *µ*l) to each sample. RNA isolation was performed with 90 *µ*l silica suspension, otherwise according to the manufacturer's protocol. RNA was eluted in 100 *µ*l NucliSENS elution buffer prior to analysis with SARS-CoV-2 RT-PCR assays.

Quantification of SARS-CoV-2 RNA by RT-PCR was performed on a LightCycler 96 (Roche Diagnostics, Norway) using the LightCycler Multiplex RNA Virus Master (Roche Diagnostics, Norway) and the following primers and probes (Eurogentec, Belgium): RdRp nCoV IP2 and IP4 [37] from Pasteur in duplex, and HKU (ORF1b-nsp14) from Chu et al. [38] in a duplex with the internal control. Probes for IP2 and IP4 used a BHQ-1 quencher instead of BBQ. Each reaction contained 4 *µ*l 5X reaction mixture, 0.1 *µ*l 200X enzyme solution, 25 *µ*g BSA, 0.5 *µ*M of each primer, 0.25 *µ*M probe, and 5 *µ*l sample. For internal control, 0.5 *µ*l primer/probe mixture was included in the master mix for the HKU assays. A final reaction volume of 20 *µ*l was reached by adding PCR-grade water. SARS-CoV-2 synthetic RNA control 1 (Twist Bioscience, CA, USA) was used as a positive control, and PCR-grade water was used as a negative control for RT-PCR. Samples were analyzed in duplicates under the following conditions: reverse transcription at 55°C for 10 min, initial denaturation at 95°C for 30 s, and finally, 45 cycles with a two-step amplification, starting with denaturation at 95°C for 5 s and annealing/extension at 58°C for 30 s.

An assay sensitivity test was performed. A Ct value of 32 was equivalent to 10^2^ copies/*μ*l for IP2/IP4, while HKU was less sensitive with a Ct value of 35 at the same concentration. 10^2^ copies/*μ*l, which amounts to a concentration of 2.2 virus copies per liter of air, was also the limit of detection (LoD) for the assays, i.e., the lowest concentration where both duplets were detected consistently. Both assays were able to detect lower concentrations in a less stable manner, with IP2/IP4 being more stable than HKU. Here, we apply the criterion that one or more RT-PCR markers Ct values must be below LoD for classifying a sample as “positive,” while samples, where aCt value above LoD was recorded, are reported as “having a Ct value.”

### 2.5. Cell Culture of Air Samples

Only samples that were RT-PCR positive (Ct value below LoD on either marker) were analyzed with cell culture assays. Vials with air filters were vortexed for 30 s before removal of the filters with sterile forceps. Fluid was extracted from the filters back into the vial as described in the RT-PCR procedure. African green monkey kidney cells (Vero E6, ATCC: CRL-1586) were used to culture the samples. Samples were incubated for 1 h at 37°C and 5% CO_2_ atmosphere before removal, and the cells were maintained in DMEM (sigma) supplemented with 5% heat-inactivated fetal bovine (FBS, Gibco) serum and 1% PSA (Gibco). The cells were propagated in a humidified 37°C incubator in an atmosphere of 5% CO_2_ for 6 days. 500 *µ*l of supernatant was collected on days 3 and 6. After RNA isolation (NucliSENS Magnetic Extraction Reagents) RT-PCR was performed as described above.

### 2.6. Viral Load Quantification in Oropharyngeal Swabs from Hospitalized Patients

Oropharyngeal swabs were collected from 12 of the 13 patients in the pilot study. For eight of the patients, oropharyngeal swabs were taken on the same day as air samples were collected (Table 1). Oropharyngeal swabs (200 *μ*L) were added to bacteriophage MS2 (Roche, Switzerland) as extraction/inhibition control and extracted using QiaAmp MinElute Virus Spin Kit (Qiagen, Germany). SARS-CoV-2 RNA real-time reverse transcriptase PCR targeting the viral envelope gene was done as described by Corman et al. [39] on the LightCycler 480 instrument (Roche). Cellular quantification in oropharyngeal samples was assessed using the cell control r-gene kit targeting the HPRT1 gene (bioMérieux, France) according to the manufacturer's instructions. Virus RNA copies were calculated using dilution series of standards calibrated against the First WHO International Standard for SARS-CoV-2 (reference standard 20/146, NIBSC, England). Viral load in oropharynx samples was determined as virus RNA copies per 1000 human cells.

### 2.7. Statistical Analyses

A Bayesian linear regression model (R; MCMCglmm: MCMCglmm [40]) was used to regress days since symptom onset on viral load from oropharyngeal samples. Default (weakly informative) priors were used. The analysis was performed with 20,000 MCMC iterations, a 5,000 burn-in, and a thinning interval of 10. Effective sample size, trace plots, and posterior distributions were used to assess model performance. 95% HDI (highest density interval) was reported for the parameter value. Significance was reported as *pMCMC*, which is two times the smaller of the MCMC estimates of the probability that a <0 or a >0, where a is the parameter value. Effect size and 95% CI was predicted with ggeffects:ggemmeans [41] for plotting.

Mean particle counts (per minute average) from three time periods were analyzed with linear models. The three-time periods are given as follows:

1. Control air sample period: a 15-minute period prior to administering NT, during which control air sampling was performed
2. NT air sample period: a 15-minute period during which NT was given, and air sampling were performed
3. NT period: a shorter period nested within the abovementioned “2. NT air sample period,” corresponding to the duration of the NT (which lasted approx, 10 minutes)

Separate analyses (R; stats:lm) were performed on each particle size bin (0.3–0.5, 0.5–1, 1–3, 3–5, 5–10, and >10 *µ*m). Reference levels were set to particle counts from the control sample. *P* values are two-sided and considered significant at <0.05.

All statistical models and visualizations were produced in R v. 4.1.1 [42].

## 3. Results

### 3.1. Air Sample RT-PCR and Cell Culture

**Figure 1:**
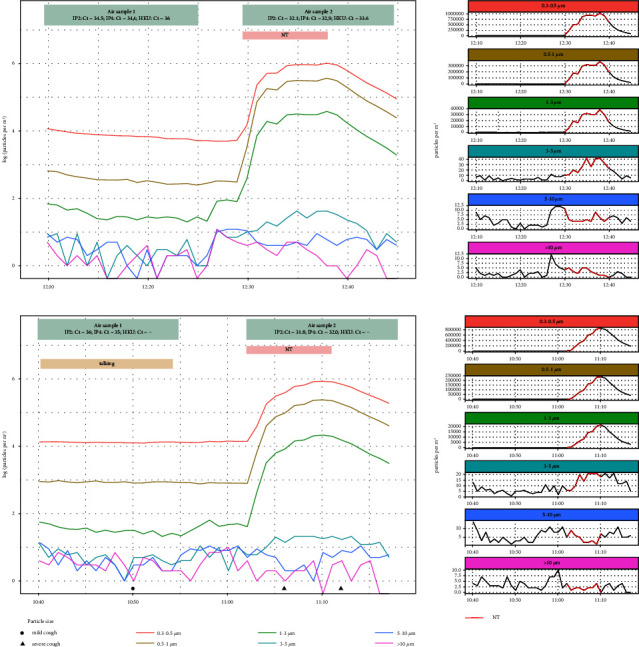
The two patients with RT-PCR positive NT air samples. Ct values and time periods for control and nebulizer therapy (NT) air samples are indicated in the top boxes of each panel. The nebulizer therapy time period is shown in pink, while continuous talking by the patient is indicated in beige. Mild and severe coughing is shown along the x-axis. The left side panels show log(e) transformed particle counts per m^3^ of air during the trials, while untransformed counts per m^3^ of air for each size bin are shown in the right side panels.

Only two air samples (from two patients) were SARS-CoV-2 positive, i.e., had one or more RT-PCR Ct values below the LoD (IP2/IP4 = 32, HKU = 35), both of which were collected during NT. The corresponding control air samples (collected before NT) for these same two patients had RT-PCR Ct values above LoD and were hence not considered positive (Table 1; Figure 1). Both patients were male, aged 50 and 61 years, and had experienced symptoms for nine and 11 days at the time of air sampling. The median for the other 11 patients was 13 days after symptom onset (Table 1).

In addition, two other patients had RT-PCR Ct values above LoD for the control air sample (Table 1) but neither of these patients had RT-PCR Ct values for the NT sample.

SARS-CoV-2 was not cultured in any of the air samples.

### 3.2. Viral Load

**Figure 2:**
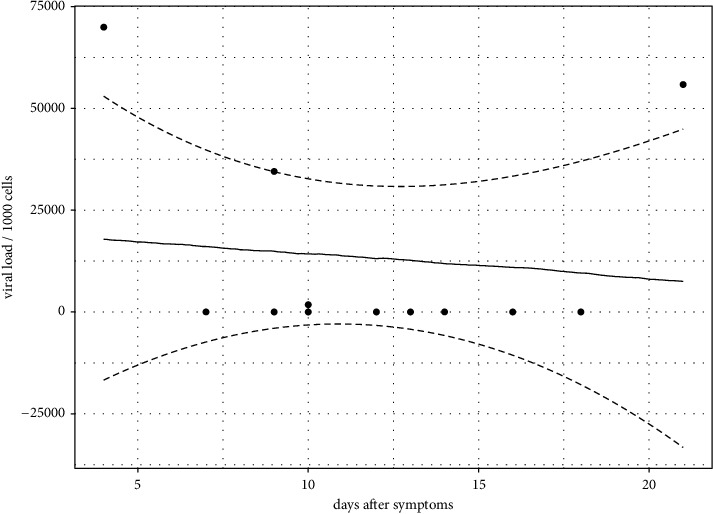
Duration of symptoms (days) regressed on viral load from oropharyngeal swab samples. Dashed lines correspond to the upper and lower limits of the 95% CI as predicted from the MCMCglmm model.

Of the 13 patients, seven had detectable virus load in their oropharyngeal sample (for one patient data was not available); three had >34,000 copies (per human 1000 cells), one had 1,780 copies, and the remaining patients had <14 copies (Table 1). Of the three patients with high viral loads (>34,000 copies), two had positive NT air samples (i.e., RT-PCR Ct value below LoD) whereas one had an RT-PCR Ct value (i.e., below LoD). There was no significant association between the duration of symptoms and viral load in oropharyngeal samples (95% HDI = [−3909.45, 3465.99], pMCMC = 0.728; Figure 2).

### 3.3. Total Particle Concentrations

**Figure 3:**
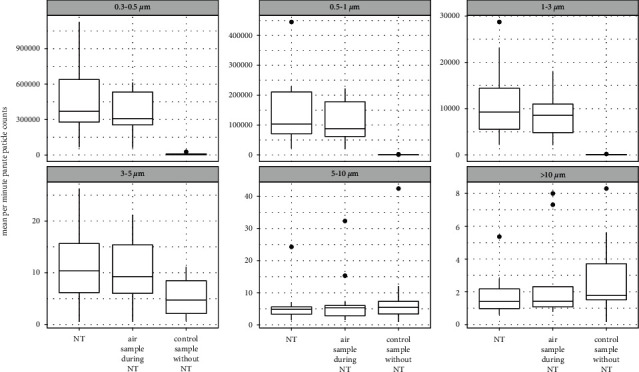
Mean (per minute) particle counts for 12 patients during the control air sample period (15 minutes), during the nebulizer therapy (NT) air sample period (15 minutes), and during the nebulizer therapy period (approx, 10 minutes).

For particle sizes 0.3–0.5, 0.5–1, and 1–3 *µ*m, concentrations were significantly higher during the NT air sampling period (15 minutes) and NT period (approx, 10 minutes; overlapping with NT air sampling period), than during the control sampling period (*p* ≤ 0.001; Table 2; Figure 3). For 3–5 *µ*m particles there were significantly higher concentrations during the NT period than during the control sample period (*p* = 0.029; Table 2), while the NT air sample period (which lasted longer than the NT period; 15 minutes) was not significant for this size bin (*p* = 0.064; Table 2). There were no significant differences in particle concentrations for particles 5–10, and <10 *µ*m (Table 2). All particle load distributions are shown in Figure 3. Analyzed data are given in Table 3.

## 4. Discussion

In this pilot study, we explored the effect of NT on the concentration of SARS-CoV-2 RNA and total particles in the air of COVID-19 patient isolation rooms. Only two air samples were positive (RT-PCR Ct values below LoD), both of which were sampled during NT. For all patients, we observed an increase in total concentrations of smaller particles (0.3–5 *µ*m) during NT.

The two patients with positive air samples were both males, which is consistent with men generally having higher disease severity [43], and prolonged viral shedding [44] compared to women. They also had shorter symptom duration than patients with RT-PCR negative air samples. This is consistent with previous studies that have demonstrated that SARS-CoV-2 viral loads in the upper respiratory tract peak during the first week of illness and are followed by a consistent decline [45]. Symptom duration and viral load in upper airways did not support such a pattern in our cohort (Figure 2); however, our analysis is limited by small sample size, only including two patients in the first week of illness and only three patients with a high viral load in the nasopharynx samples (Table 1).

As could be expected, we observed that patients with high viral load in the oropharynx disseminated more viral RNA into the air; the three patients with high viral loads (>34,000 copies per cell) included the two patients that had RT-PCR positive air samples collected during NT and one additional patient, whose control air sample (collected prior to NT) had an IP4 RT-PCR Ct value of 37.66. Of note, not all air and oropharynx samples were collected on the same day, which precluded direct comparisons for some samples. Finally, one patient had RT-PCR IP2/4 Ct values for the air sample collected prior to NT, and a viral load of zero copies in the oropharynx sample (samples were collected on the same day). This incongruity may be explained by upper respiratory sampling not being optimal for detecting lower respiratory tract infection [46], i.e., viral RNA in the air may have been disseminated from the lower respiratory tract, even though viral levels in the upper tract were too low for detection. The chosen patient sampling methodology, namely, oropharyngeal, is also less sensitive than combined nasopharynx/oropharynx sampling, which likely negatively affected detection levels in the patient samples [47].

Four patients had control sample RT-PCR Ct values above LoD. These included the two patients with positive NT air samples, but also two other patients with no Ct values for their NT air samples. Of these latter two patients, one had a viral load of >50,000, while the other had a viral load of zero (Table 1); however, as mentioned in the previous paragraph, the patient with a viral load of zero may have had an infection in the lower respiratory tract. That Ct values were recorded for control samples and not NT samples can be due to viral concentrations in air being below LoD in both cases. Alternatively, the control sample Ct values may stem from room contamination or cross-contamination in the laboratory.

In our analysis, we opted to treat the three SARS-CoV-2 PCR markers separately, even though the three were run on all samples in parallel. There were two reasons for this approach: firstly, given the dilution effect in air and the high air exchange rate inside the isolation rooms, we expected the results to be near the LoD of the RT-PCR assays; secondly, different markers have different sensitivity—our air sample results demonstrated similar results to that reported for patient samples, with RdRp-IP2 and RdRp-IP4 appearing more sensitive than HKU [48].

The RT-PCR assay sensitivity test indicated a LoD of 2.2 virus copies per liter of air. Some other SARS-CoV-2 air studies report lower LoDs [49, 50]; however, while many studies do not report LoD in terms of viral concentrations in air, the lowest reported values across several SARS-CoV-2 air sampling studies indicate large variations in LoD [51].

Only two samples met the criteria for cell culture, which was a below LoD RT-PCR Ct value on any marker, but neither of these two samples were culture positive. This may be because replication-competent virus was not present in the samples; the patients in our pilot study had been ill for a long period of time and detectable virus may simply reflect remnant RNA and not infectious virus [45]. Isolation of infectious viruses has only rarely been possible from upper respiratory specimens later than 10 days after the start of symptoms [52]. Alternatively, loss of viability may have been inflicted during air sampling. Using a condensation growth air sampling method, that is potentially more gentle, Lednicky et al. [53] cultivated SARS-CoV-2 from a sample collected at 4.8 meters from one hospitalized COVID-19 patient; the patient in question had suffered from respiratory illness for only two days when air sampling was performed, which may also explain why SARS-CoV-2 was successfully cultured in this case.

Whether NT constitutes an AGP is a topic of contention [18, 19, 29–31, 33–35], which needs to be resolved given the potential implications for HCW safety. Clearly, NT generates aerosols by changing liquid medication to mist, but in medical terminology AGP implies the generation of aerosols from potentially infectious bodily fluids and tissues. In our pilot study, we were unable to demonstrate that SARS-CoV-2 RNA in the air during NT was from replication-competent virus. However, our results support the notion that NT may increase the dissemination of viral RNA from patient tissues and that aerosol particles from NT do not only come from aerosolized medication. Therefore, we recommend that HCW use airborne precautions when administering NT to patients, including filtering facepiece respirators equivalent to N95/FFP2 or higher, especially during the first period of illness when the patient is most likely to be infectious. Alternative methods for administrating aerosolized medication, that is likely to cause less contamination of the air, such as valved holding chambers, i.e., spacers with one-way valves that contain aerosol particles until inspiration occurs, may reduce the risk of transmission to HCW.

Our pilot study is limited by the relatively low number of air samples collected from patients with rather a long duration of COVID-19 and the low number of RT-PCR positive air samples. Furthermore, although sampling was performed before and during NT, the data are based on only two sampling points per patient and do not consider potential within-patient variability. The limited number of positives is likely due to the patient group, on average, being in the later stages of the disease, where viral loads in respiratory tissues decline [45]. Furthermore, it is likely that the high air exchange rate played a part in rapidly reducing aerosol concentrations of SARS-CoV-2 RNA in the isolation rooms where the pilot study was carried out [54]. Given the low concentrations of viral RNA in air, we recommend that future studies sample for longer time periods than was used in the present pilot study.

We have shown that NT is associated with increased total aerosol particle concentrations for particles of sizes 0.3–5 *µ*m. Although the dataset collected in this pilot study can only give preliminary information about the effect of NT, for two patients, we found SARS-CoV-2 RNA concentrations to increase in the surrounding air during NT. This indicates that aerosols associated with NT are not simply a product of the administered medication but may also originate from the patient's bodily fluids and tissues. Therefore, NT may involve an increased risk of infection for HCW. Given the importance of understanding the risks associated with NT, we recommend that similar studies be carried out with larger groups of patients that are at earlier stages of COVID-19.

## Figures and Tables

**Table 1:** All analyzed data.

			Air samples																		Oropharyngeal swabs		
Age	Sex	Date of symptom onset	Date of sample collection	Days after symptom onset	Potential aerosol-generating behavior during control sample period (before NT)^*∗∗*^					Potential aerosol-generating behavior during NT sample period^*∗∗*^					Control sample (before NT)			NT sample			Date of sample collection	Days after symptom onset	Viral load/1000 cells
					Talking	Mild cough	Severe cough	Laughing	Sneeze	Talking	Mild cough	Severe cough	Laughing	Sneeze	Ct-IP2	Ct-IP4	Ct-HKU	Ct-IP2	Ct-IP4	Ct-HKU			
35–40	Female	14.10.2020	06.11.2020	23	0	0	0	0	0	1	0	0	0	0	—	—	—	—	—	—	28.10.2020	14	13
65–70	Male	19.11.2020	30.11.2020	11	3	0	0	0	0	0	0	0	0	0	—	—	—	—	—	—	29.11.2020	10	1 780
60–65	Male	19.11.2020	30.11.2020	11	15	1	0	0	0	0	0	2	0	0	36^*∗*^	35^*∗*^	—	31.81 ± 0.02	31.97 ± 0.06	—	23.11.2020	4	69 942
50–55	Male	11.11.2020	30.11.2020	19	15	0	0	0	0	1	1	0	0	0	—	—	—	—	—	—	—	—	—
45–50	Male	02.01.2021	11.01.2021	9	15	0	0	0	0	4	11	0	0	0	35.79	36, 83 ± 1, 46	—	—	—	—	11.01.2021	9	0
80–85	Male	01.01.2021	11.01.2021	10	15	0	0	0	0	7	0	0	0	0	—	—	—	—	—	—	11.01.2021	10	0
50–55	Female	04.01.2021	11.01.2021	7	0	0	0	0	0	0	0	0	0	0	—	—	—	—	—	—	11.01.2021	7	13
55–60	Male	07.01.2021	20.01.2021	13	15	0	0	0	0	0	0	0	0	0	—	—	—	—	—	—	20.01.2021	13	3
40–45	Male	04.01.2021	20.01.2021	16	15	0	0	0	0	0	0	0	0	0	—	—	—	—	—	—	20.01.2021	16	0
60–65	Female	02.01.2021	20.01.2021	18	0	0	0	0	0	0	0	0	0	0	—	—	—	—	—	—	20.01.2021	18	0
50–55	Male	18.01.2021	27.01.2021	9	0	0	0	0	0	0	0	0	0	0	34, 46 ± 0, 94	34, 38 ± 0, 13	36^*∗*^	32, 13 ± 0, 55	32, 87 ± 0, 25	33.61	27.01.2021	9	34 515
55–60	Female	15.01.2021	27.01.2021	12	15	0	0	0	0	0	0	0	0	0	—	—	—	—	—	—	27.01.2021	12	0
60–65	Female	11.01.2021	27.01.2021	16	0	0	0	0	0	0	0	0	0	0	—	37.66	—	—	—	—	01.02.2021	21	55 854

^*∗*^Manual reading of amplification curve. ^*∗∗*^Talking is measured in minutes, while the other behaviors are counted.

**Table 2:** Linear models comparing mean (per minute) particle counts during the control air sample period (15 minutes) with counts during the nebulizer therapy (NT) air sample period (15 minutes) and the nebulizer therapy period (approx, 10 minutes). Particle counts from the control air samples were set as the reference levels in the models.

	Predictors	Estimates	CI	*P*
0.3–0.5 *µ*m	(Intercept)	7010.29	−110238.05–124258.64	
	NT air sample period	352655.80	186841.61–518470.00	<0.001
	NT period	440291.92	274477.72–606106.11	<0.001
	Observations	36		
	*R * ^2^/*R*^2^ adjusted	0.498/0.467		

0.5–1 *µ*m	(Intercept)	525.00	−44487.51–45537.50	
	NT air sample period	109909.43	46252.14–173566.72	0.001
	NT period	143178.80	79521.51–206836.09	<0.001
	Observations	36		
	*R * ^2^/*R*^2^ adjusted	0.410/0.374		

1–3 *µ*m	(Intercept)	61.20	−3317.85–3440.26	
	NT air sample period	9027.26	4248.55–13805.97	0.001
	NT period	11636.99	6858.29–16415.70	<0.001
	Observations	36		
	*R * ^2^/*R*^2^ adjusted	0.450/0.417		

3–5 *µ*m	(Intercept)	5.47	1.57–9.37	
	NT air sample period	5.20	−0.31–10.72	0.064
	NT period	6.183	0.67–11.70	0.029
	Observations	36		
	*R * ^2^/*R*^2^ adjusted	0.154/0.103		

5–10 *µ*m	(Intercept)	8.48	3.31–13.66	
	NT air sample period	−0.99	−8.31–6.32	0.784
	NT period	−2.49	−9.80–4.83	0.494
	Observations	36		
	*R * ^2^/*R*^2^ adjusted	0.014/−0.045		

>10 *µ*m	(Intercept)	2.77	1.54–4.00	
	NT air sample period	−0.29	−2.03–1.45	0.738
	NT period	−0.94	−2.68–0.80	0.280
	Observations	36		
	*R * ^2^/*R*^2^ adjusted	0.037/−0.021		

**Table 3:** Mean (per minute) particle counts for 12 patients during the control air samples (15 minutes), during the nebulizer therapy (NT) air samples (15 minutes), and during nebulizer therapy (approx, 10 minutes).

Id	Sample type	0.3–0.5 *µ*m	0.5–1 *µ*m	1–3 *µ*m	3–5 *µ*m	5–10 *µ*m	>10 *µ*m
1	Control air sample	13067	869	34	5	4	4
1	NT air sample	444579	115319	9876	15	5	2
1	NT	429120	114387	9994	15	4	2
2	Control air sample	26815	2312	119	11	8	4
2	NT air sample	201995	49507	4271	10	5	1
2	NT	222613	55080	4776	10	6	1
3	Control air sample	7571	376	34	3	3	2
3	NT air sample	538017	178115	17476	21	5	2
3	NT	683162	230186	22922	26	6	3
4	Control air sample	1279	156	64	10	6	2
4	NT air sample	529887	177779	18094	21	6	2
4	NT	623659	216669	23176	26	6	3
5	Control air sample	3349	332	38	1	5	1
5	NT air sample	148372	57216	4409	1	4	1
5	NT	156288	59386	4501	1	4	1
6	Control air sample	4669	352	207	8	42	8
6	NT air sample	612392	179035	9988	9	32	7
6	NT	704360	208721	11567	7	24	5
7	Control air sample	5214	359	52	2	12	6
7	NT air sample	603217	221854	13962	2	15	8
7	NT	1125716	444965	28720	2	5	2
8	Control air sample	642	60	7	1	1	0
8	NT air sample	286782	87737	8489	9	2	1
8	NT	342058	101785	9526	11	1	1
9	Control air sample	4149	276	29	4	3	2
9	NT air sample	272819	63652	4984	7	3	1
9	NT	317568	74987	5824	7	3	1
10	Control air sample	820	84	11	2	2	1
10	NT air sample	286462	87245	8652	17	2	1
10	NT	297897	91247	9011	17	2	1
11	Control air sample	4361	423	66	8	7	2
11	NT air sample	62827	20787	2130	4	3	1
11	NT	65840	21696	2205	4	3	1
12	Control air sample	12187	700	73	10	7	2
12	NT air sample	328644	86969	6731	14	7	1
12	NT	399344	105335	8157	14	7	1

## Data Availability

The analyzed data are presented in Tables 1 and 3.

## Additional Points

*Practical Implications*. In this pilot study, we found that nebulizer therapy increased the concentration of smaller particles (0.3–5 *µ*m) and SARS-CoV-2 RNA in the air surrounding COVID-19 patients receiving such treatment. This is consistent with the notion that nebulizer therapy may increase the risk of exposure to SARS-CoV-2 for health care workers treating patients with COVID-19. Our results support the use of precautions against airborne infections when administering nebulizer therapy and that, if possible, less aerosol-producing nebulizer treatment modalities should be used. Furthermore, research is warranted to determine whether nebulizer therapy may increase the risk for transmission of replication-competent SARS-CoV-2.
